# Propriety of various examinations for subjective symptoms of olfactory disorders

**DOI:** 10.1007/s00405-024-08803-w

**Published:** 2024-06-28

**Authors:** Tomotaka Hemmi, Kazuhiro Nomura, Yuta Kobayashi, Yuki Numano, Ryoukichi Ikeda, Mitsuru Sugawara

**Affiliations:** 1 Department of Otolaryngology, Tohoku Kosai Hospital, 2-3-11 Kokubun-cho, Aoba-ku, Sendai, 980-0803 Japan; 2 Department of Otolaryngology, Head and Neck Surgery, Tohoku University School of Medicine, 1-1 Seiryo-Machi, Aoba-ku, Sendai, 980-8574 Japan; 3 Department of Otolaryngology-Head and Neck Surgery, Iwate Medical University School of Medicine, 1-1-1 Yahaba-cho, Shiwa-gun, Iwate, 028-3695 Japan

**Keywords:** CT, Examination, Intravenous olfactory test, Olfactory disorder, T&T olfactometry

## Abstract

**Purpose:**

In Japan, two types of tests for diagnosing olfactory disorders, T and T (T&T) olfactometry and intravenous olfactory tests, are covered by insurance and performed on patients with olfactory disorders. This study examined the validity of these olfactory tests and whether psychophysical or morphological tests are more helpful in evaluating olfactory disorders.

**Methods:**

We evaluated patients who visited our department and underwent two types of olfaction tests and sinus computed tomography (CT). Data regarding the age, sex, peripheral blood eosinophil percentage, presence of bronchial asthma, diagnoses, olfactory symptom score, results of the two olfactory tests, and CT findings in eligible patients were extracted from medical records and retrospectively reviewed.

**Results:**

One hundred and sixty-three patients underwent all tests during the study period. The results of the T&T olfactometry and intravenous olfactory tests were significantly correlated. However, only the results of T&T olfactometry and olfactory cleft opacification on CT were statistically significant predictors of the olfactory symptom scores.

**Conclusion:**

T&T olfactometry and CT evaluations of olfactory cleft opacification helped evaluate olfactory dysfunction. It is important to note that intravenous olfactory tests are best performed with careful control and not blindly to assess olfactory disorders.

## Introduction

In Japan, two types of tests for diagnosing olfactory disorders, T and T (T&T) olfactometry and intravenous olfactory tests, are covered by insurance and performed on patients with olfactory disorders. T&T olfactometry is an orthonasal test that involves sniffing five types of odorants and quantifying their detection and recognition thresholds [1]. The intravenous olfactory test is performed with proslutiamine and is a retronasal test, evaluating the time from injection into an antecubital vein until the odor is perceived as the latency and the time until the odor is no longer felt as the duration [2]. These two tests are subjective and psychophysical, and we often experience a disconnect between the patient's chief complaint and the test results. In addition, an intravenous olfactory test may not be able to be performed because of pain or mood discomfort. Opacification of the ethmoid sinus and olfactory cleft is associated with olfactory dysfunction [3]. Although they can be evaluated objectively by computed tomography (CT), performing all of these examinations is also considered undesirable from a cost-efficiency standpoint.

The present study examined the validity of the two types of olfactory tests and determined the most effective psychophysical or morphological tests for evaluating olfactory disorders.

## Material and methods

### Subjects

We evaluated patients who visited the Otolaryngology Department of Tohoku Kosai Hospital in Japan between December 2021 and October 2022 and underwent two types of olfaction tests and sinus CT. Data regarding the age, sex, peripheral blood eosinophil percentage, presence of bronchial asthma, diagnoses, and olfactory symptom score (0, no problem; 1, very mild problem; 2, mild or slight problem; 3, moderate problem; 4, severe problem; 5, problem as bad as it can be) were extracted from medical records and retrospectively reviewed.

This study was approved by the Ethics Committee of Tohoku Kosai Hospital (kkrtohoku-202307otor_S1-1_01).

### Olfactory tests

T&T olfactometry was performed using the following five odorants: (A) β-phenylethyl alcohol, which smells like a rose; (B) methyl cyclopentenolone, which smells like burning sugar; (C) isovaleric acid, which smells like sweat; (D) γ-undecalactone, which smells like canned peaches; and (E) skatole, which smells like excrement (Daiichi Yakuhin Sangyo Company Limited, Tokyo, Japan). The concentration range for each odorant covered 8° of intensity (− 2–5), except for odorant B, which covered 7° (− 2–4). The detection and recognition thresholds were determined for each odorant.

An intravenous olfactory test was performed using proslutiamine (Takeda Pharmaceutical Company Ltd., Osaka, Japan). Proslutiamine (10 mg, 2 ml) was injected into the antecubital vein at an even rate over 20 s. The latency was defined as the first report of a definite garlic or onion smell from the start of the injection. The duration was defined as the time from the first perception of the odor to its disappearance. In all cases, olfactory tests were performed on the same day with a time delay.

### CT findings

CT images were primarily obtained using a Brilliance CT 64 (Royal Philips, Amsterdam, Netherlands) with a thickness of 2 mm per slice. For the evaluation of CT findings, the left and right olfactory clefts and ethmoid sinuses were assessed on a scale of 0–2 (0, no abnormality; 1, partially opacified; and 2, completely opacified) by a board-certified otorhinolaryngologist in a blinded manner. The olfactory cleft was defined as the distance between the superior turbinate and the nasal septum. In most cases, the CT and aforementioned olfactory examinations were performed within a month's interval.

### Statistical analyses

All values are expressed as mean ± standard deviation (SD). A Pearson correlation analysis was used to examine these associations. A logistic single regression analysis was used to evaluate the nominal scale. In addition, a logistic multivariate regression analysis was used to examine the items related to the perception of olfactory disorders.

All analyses were performed using R version 4.2.2. Statistical significance was set at P < 0.05.

## Results

### Subjects’ characteristics

During the study period, 163 patients underwent all tests described above. The demographic data of the patients are presented in Table 1. Almost all patients (93.9%) had chronic rhinosinusitis (CRS) (93.9%). This study registered sinus fungus balls and odontogenic maxillary sinusitis as CRS cases. Patients with post-traumatic and post-infectious olfactory dysfunction were not observed in this study. The most common olfactory symptom score was 0 (29.4%), followed by 5 (20.8%). The CT scores were approximately equal between the olfactory and ethmoid sinuses.

**Table 1:** Patients’ information, diagnoses, symptoms, and CT scores

Item	Value
Patients (n)	163
Age (years)	51.8 ± 15.3
Male/female	104/59
Peripheral blood eosinophil (%)	5.3 ± 4.6
Presence of bronchial asthma Y/N	36/127
Diagnosis (number of patients)	
Chronic rhinosinusitis	153
Hypertrophic rhinitis	84
Deflected nasal septum	68
Inverted papilloma	10
Allergic rhinitis	4
Allergic fungal rhinosinusitis	1
Invasive fungal rhinosinusitis	1
Symptom score	
0: No problem	48
1: Very mild problem	22
2: Mild or slight problem	19
3: Moderate problem,	17
4: Severe problem	23
5: Problem as bad as it can be	34
CT score	
Olfactory cleft (0–4)	1.6 ± 1.7
Ethmoid sinus (0–4)	1.7 ± 1.0

Diagnosis is indicated with duplicates

### Correlation of the two olfactory tests

**Fig. 1:**
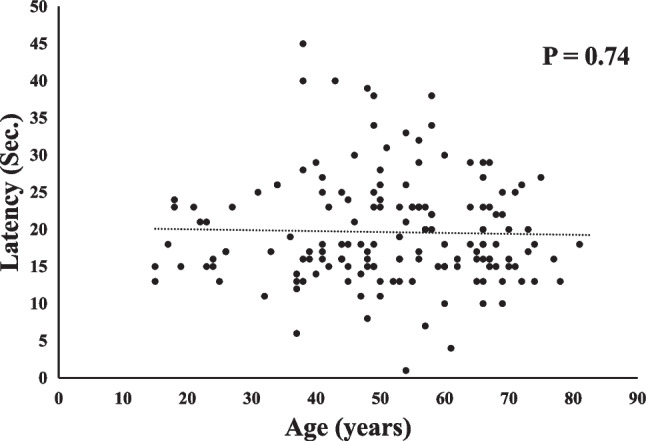
No statistically significant correlation was found between the age and the latency of intravenous olfactory test (P = 0.74)

**Fig. 2:**
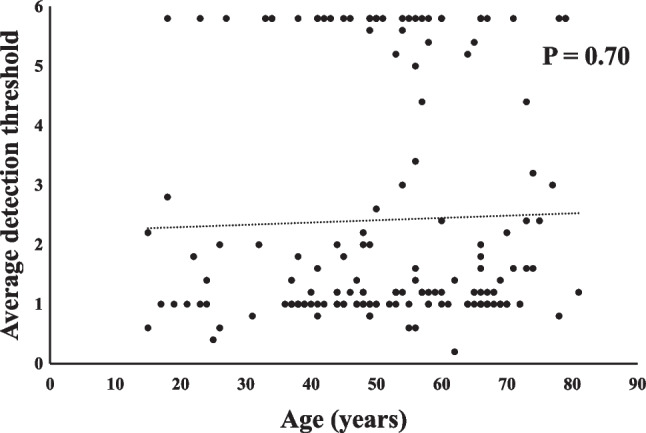
No statistically significant correlation was found between the age and the average detection thresholds of five odorants in T and T (T&T) olfactometry (P = 0.70)

Details of the two olfactory test results are presented in Table 2. No significant correlation was found between age and the two olfaction tests (Figs. 1, 2). The latencies and durations obtained from the intravenous olfactory test were significantly correlated with the detection and recognition thresholds for each odorant obtained via T&T olfactometry (Table 3). Patients who were unresponsive to the intravenous olfactory test were excluded from the analysis.

**Table 2:** Summary of olfactory test findings (N = 163)

Item	Value
Intravenous olfactory test	
Non-responsive cases (n)	8
Latency (s)	19.6 ± 7.2
Duration (s)	60.7 ± 28.1
T&T test	
Detect A (-2-5)	2.8 ± 2.1
Detect B (-2-4)	2.2 ± 1.7
Detect C (-2-5)	2.2 ± 2.1
Detect D (-2-5)	2.4 ± 2.1
Detect E (-2-5)	2.3 ± 2.1
Recognize A (-2-5)	3.4 ± 2.0
Recognize B (-2-4)	2.4 ± 1.7
Recognize C (-2-5)	2.3 ± 2.2
Recognize D (-2-5)	2.9 ± 2.1
Recognize E (-2-5)	2.6 ± 2.1

**Table 3:** Correlation of olfactory tests

	T&T test	Correlation coefficient	p value		T&T test	Correlation coefficient	P value
Latency of intravenous olfactory test	Detect A	0.43	< 0.001	Duration of intravenous olfactory test	Detect A	− 0.29	< 0.001
	Detect B	0.51	< 0.001		Detect B	− 0.25	0.001
	Detect C	0.51	< 0.001		Detect C	− 0.26	< 0.001
	Detect D	0.50	< 0.001		Detect D	− 0.26	< 0.001
	Detect E	0.50	< 0.001		Detect E	− 0.27	< 0.001
	Recognize A	0.38	< 0.001		Recognize A	− 0.23	0.003
	Recognize B	0.46	< 0.001		Recognize B	− 0.23	0.003
	Recognize C	0.51	< 0.001		Recognize C	− 0.27	< 0.001
	Recognize D	0.45	< 0.001		Recognize D	− 0.20	< 0.001
	Recognize E	0.45	< 0.001		Recognize E	− 0.60	< 0.001

### Relationship between olfactory symptom score and various examination

Table 4 shows the results of the analysis of the two olfaction test findings, assuming that patients with an olfactory symptom score of 0 or 1 were in the normal olfaction group, while patients with an olfactory symptom score of 4 or 5 were in the abnormal olfaction group. A univariate analysis showed that all olfactory test results, except for the durations obtained from the intravenous olfactory test, were significantly predictive of the presence of olfactory disorders.

**Table 4:** Results of a univariate analysis of olfactory disorder symptoms and olfaction test findings

		Symptom 0–1 (n = 69)	Symptom 4–5 (n = 54)	Odds ratio	p value
Intravenous olfactory test	Latency (s)	17.1 ± 5.6	23.7 ± 7.3	1.1	< 0.001
	Duration (s)	62.8 ± 29.1	58.2 ± 25.0	0.9	0.197
T&T test	Detect A (-2-5)	1.6 ± 1.2	4.1 ± 2.0	1.9	< 0.001
	Detect B (-2-4)	1.2 ± 0.8	3.2 ± 1.8	2.5	< 0.001
	Detect C (-2-5)	1.0 ± 0.9	3.5 ± 2.5	2.0	< 0.001
	Detect D (-2-5)	1.2 ± 0.9	3/7 ± 2.2	2.1	< 0.001
	Detect E (-2-5)	1.2 ± 0.9	3.6 ± 2.4	1.9	< 0.001

To examine in more detail the factors that predict the presence or absence of olfactory dysfunction, a multivariate analysis was performed, including the age, sex, olfactory cleft and ethmoid sinus scores on CT, and peripheral blood eosinophil percentage, in addition to the olfactory test results. The latencies obtained from the intravenous olfactory test did not suggest the presence of olfactory disorders (Table 5). In contrast, thresholds for odorant B in the T&T olfactometry were a significant factor indicating the presence of olfactory disorders (Table 6). In addition, the olfactory cleft score on CT was a significant indicator of olfactory disorders. Patients who were unresponsive to the intravenous olfactory test were excluded from the analysis.

**Table 5:** Results of a multivariate analysis of olfactory disorder symptoms and intravenous olfactory test

Item	Odds ratio	95% CI	p value
Latency	1.0	0.9–1.1	0.343
Age	0.9	0.9–1.0	0.845
Sex	0.4	0.1–1.4	0.199
OC score	1.9	1.2–3.3	0.007*
ES score	2.9	1.3–7.3	0.011*
Eosinophil	1.0	0.9–1.2	0.250

*CI* confidence interval, *OC* olfactory cleft, *ES* ethmoid sinus

*p value < 0.05

**Table 6:** Results of a multivariate analysis of olfactory disorder symptoms and T&T test findings

Item	Odds ratio	95% CI	p value
Detect B	1.8	1.3–2.9	0.001*
Age	0.9	0.9–1.0	0.528
Sex	0.7	0.2–2.4	0.596
OC score	1.8	1.0–3.2	0.028*
ES score	1.9	0.8–5.0	0.148
Eosinophil	1.0	0.9–1.2	0.197

*CI* confidence interval, *OC* olfactory cleft, *ES* ethmoid sinus

*p value < 0.05

## Discussion

This study revealed that, while T&T olfactometry is helpful in assessing olfactory dysfunction, intravenous olfactory tests cannot evaluate olfactory dysfunction with the same accuracy as T&T olfactometry. In addition, intravenous olfactory tests are invasive, and their indications should be thoroughly assessed.

Intravenous olfactory tests help estimate the degree of olfactory disorders, differentiate impaired lesions, and predict the olfactory prognosis, with a latency of ≤ 10 s and a duration of ≥ 1 min, which is considered normal. Non-response to this test suggests irreversible olfactory disorders [2]. In animal studies, Kikuta et al. found that a prolonged latency was suggestive of olfactory neuron damage and depletion and that a prolonged latency in patients with CRS was associated with a poor prognosis for postoperative improvement of the olfactory function [4]. Based on the results of our study and previous studies, we believe that intravenous olfactory tests should not be performed blindly to assess olfactory disorders but rather to estimate the mechanism underlying the symptoms and the prognosis of olfactory disorders.

Animal studies have shown that the five odorants used in T&T olfactometry activate a large number of glomeruli, and the distribution pattern of activated glomeruli is specific to each odorant [5]. Studies in Japanese and Korean patient populations have reported that such an approach is helpful for the assessment of olfactory disorders [6, 7]. In the present study, responses to all odorants used in T&T olfactometry were significantly associated with perceived olfactory disorders. Since the test is noninvasive and relatively easy to perform, it should be considered the first test to evaluate the severity of olfactory dysfunction and determine the effectiveness of treatment. Compared to other odorants, especially the decreased response to odorant B suggested olfactory disorders. It has also been reported that olfactory event-related potentials to odorant B differ between healthy adults and patients with olfactory dysfunction [8]. The response to odorant B could be a useful indicator in assessing olfactory dysfunction.

Using surgical specimens, Omura et al. demonstrated that the olfactory epithelium is selectively located in the anterior two-thirds of the superior turbinate and the nasal septum opposite the superior turbinate [9]. There are scattered reports that opacification of the olfactory cleft is significantly correlated with perceived olfactory dysfunction [10–12], and the same was true in the present study. It is important to remember that subjective examinations and imaging evaluations are crucial for an accurate understanding of olfactory dysfunction, and CT is beneficial for assessing the olfactory cleft [13].

CRS is divided into CRS with nasal polyps (CRSwNP) and CRS without nasal polyps (CRSsNP) according to the presence or absence of nasal polyps, and for CRSwNP, especially eosinophilic CRS (ECRS), the blood eosinophilia (> 5%) is included in the diagnostic criteria [14]. Although patients with ECRS have been reported to have more severe olfactory disturbances [3], eosinophil rates in peripheral blood were not a predictive factor for symptom scores in this study. Because of the variety of endotypes of CRS, the search for a common biomarker that can predict olfactory dysfunction due to CRS is not easy.

Several limitations associated with the present study warrant mention. First, it was a retrospective study conducted at a single institution. The possibility that different conclusions can be drawn from other racial and patient groups cannot be ruled out. However, as CRS is a common cause of olfactory dysfunction [15], the patient population in this study was appropriate. Second, the superior turbinate is known to be pneumatized at a rate of 7.1% [16]. The possibility that the narrowing of the olfactory cleft by pneumatized superior turbinate may affect the accuracy of CT readings of the olfactory cleft cannot be completely ruled out. Third, the relationship between the severity of olfactory disorders and the results of various tests has not been assessed. This study evaluated olfactory symptom scores of 0 and 1 as indicating no olfactory disorders and 4 and 5 as indicating olfactory disorders to simplify the statistics and obtain an odds ratio. Although cases with olfactory symptom scores of 2 and 3 were not included in the study, the number of patients was still sufficient for a statistical analysis, which helped interpret the results. In addition, cases unresponsive to intravenous olfactory tests were excluded from the analysis. However, this approach avoids arbitrary results.

## Conclusion

T&T olfactometry and CT evaluations of olfactory cleft opacification proved helpful for evaluating olfactory dysfunction. It is important to note that intravenous olfactory tests are best performed with careful control and not blindly to assess olfactory disorders.
